# Nutrition and Glycemic Control in Children and Adolescents with Type 1 Diabetes Mellitus Attending Diabetes Camps

**DOI:** 10.3390/nu16193338

**Published:** 2024-10-01

**Authors:** Kleoniki I. Athanasiadou, Maria Papagianni, Theodora Psaltopoulou, Stavroula A. Paschou

**Affiliations:** 1 Endocrine Unit and Diabetes Centre, Department of Clinical Therapeutics, Alexandra Hospital, School of Medicine, National and Kapodistrian University of Athens, 115 28 Athens, Greece; kleoniki.ath@gmail.com (K.I.A.); tpsaltop@hotmail.com (T.P.); 2 Department of Nutrition and Dietetics, School of Physical Education, Sport Science and Dietetics, University of Thessaly, 421 32 Trikala, Greece; marpapagianni@hotmail.com; 3 Endocrine Unit, 3rd Department of Pediatrics, Hippokration Hospital of Thessaloniki, Aristotle University of Thessaloniki, 546 42 Thessaloniki, Greece

**Keywords:** diabetes camps, T1DM, nutrition, glycemic control, children, adolescents

## Abstract

**Background/Objectives:** Diabetes camps for children and adolescents with Type 1 Diabetes mellitus (T1DM) offer the opportunity to have a camping experience in a safe and supportive environment where they can receive diabetes skills education, such as glucose self-monitoring, insulin injections, management of hypoglycemia/hyperglycemia, and nutritional recommendations, including meal planning and carbohydrate counting. The ultimate goal of diabetes camps is to educate children to manage their condition independently, without parental involvement. Additionally, attending a diabetes camp is an excellent opportunity to meet peers and share their experiences and concerns about their condition, enhancing their confidence and reducing diabetes-related emotional distress. The aim of this review was to assess whether the nutritional planning and education offered at diabetes camps has a favorable effect on the glycemic control of attending children and adolescents. **Methods:** A literature search in PubMed and Scopus databases was performed. Eligible for inclusion were studies evaluating the effect of nutritional education offered in diabetes camps on glycemic control of children and adolescents with T1DM. **Results:** The majority of identified eligible studies supported the beneficial impact of the nutritional education offered in diabetes camps on glycemic control during and after the camp sessions. The favorable effect, though, seemed to be temporarily sustained (<6 months). **Conclusions:** Continuous nutritional education is required to prolong the duration of these beneficial outcomes. Further interventional studies are required to evaluate the direct effect of nutritional education provided at diabetes camps on glycemic control of children and adolescents with T1DM and the actual duration of favorable outcomes.

## 1. Introduction

Type 1 diabetes mellitus (T1DM) is a potentially life-threatening chronic autoimmune disease characterized by complete insulin deficiency resulting in hyperglycemia. It is commonly diagnosed during childhood or adolescence, usually presenting as diabetic ketoacidosis (DKA) [1,2]. Upon diagnosis, patients are commonly insulin deficient and are put on insulin replacement treatment, initially with multiple daily injections. It is essential that healthcare professionals provide intensive initial education for T1DM (blood glucose monitoring, insulin injections, dosing titration in special circumstances, management of hypoglycemia, carbohydrate counting, dietary and lifestyle modifications, etc.). Regular medical follow-up to standardize insulin doses and monitor glycemic control is required [3,4]. Psychological support to patients and their families is crucial, too, as a T1DM diagnosis can be emotionally distressing and a major challenge for family well-being [5]. Patients should be encouraged to express their concerns and receive ongoing support from their healthcare providers and community services. Daily insulin injections, a nutritious diet, and regular physical exercise are the mainstays of achieving optimal glycemic control and avoiding T1DM complications (hypoglycemia, DKA, microvascular and macrovascular complications) [6,7]. The first diabetes camp opened in 1925 in Michigan (USA), founded by Dr Leonard F.C. Wendt. It was the first disease-specific camp in history, introduced a few years after the discovery of insulin [8]. Today, diabetes camps have become an integral part of diabetes care, offering an excellent opportunity for children and adolescents with T1DM to learn their condition better and become familiarized with the lifestyle modifications required [9,10]. The nutritional teaching and meal planning during the camping days aspire to positively affect the future dietary choices of children and adolescents with T1DM.

The aim of the present review was to assess the effect of nutritional education offered in diabetes camps on the glycemic control of attending children and adolescents with T1DM. To the best of our knowledge, this is the first review evaluating the influence of the nutritional education provided in diabetes camps on the glycemic control of children and adolescents with T1DM.

## 2. Search Strategy

A literature search was conducted in PubMed and Scopus from inception until August 2024 using combinations of the key words: “diabetes camps,” “nutrition*,” “nutritional education,” “diet,” “dietary planning,” “glycemic control,” “glycated hemoglobin,” “type 1 diabetes mellitus,” and “T1DM.” Eligible for inclusion were all types of studies written in the English language.

## 3. Nutritional Education in Diabetes Camps

Diabetes camps provide a structured schedule that includes nutritional education, meal planning, sports, and social activities. Several educational sessions are addressed, including blood glucose monitoring, insulin injections, nutritional education, carbohydrate counting, and recognizing the signs and symptoms of hyperglycemia and hypoglycemia [11]. The presence of specially trained staff and a medical team consisting of physicians, diabetes nurses, dietitians, and psychologists forms a protected care setting for diabetes education and communication between participants.

Additionally, in diabetes camps, children and adolescents with T1DM have the opportunity to meet peers with their condition and share their stories, have a camping experience in a safe environment under medical surveillance, and achieve independent management of T1DM without parental involvement, developing personal responsibility and consciousness [12,13]. Goal-setting for a specific self-management skill or a diabetes-related educational topic before camping attendance can be very effective, as the participant will have the opportunity to focus and work toward a precise goal which, when achieved, will increase independence in diabetes care and enhance self-confidence [14,15]. According to studies, the quality of life and psychosocial health of children and adolescents having attended a diabetes camp is significantly improved compared to those who have not [16,17,18].

The nutritional recommendations for children and adolescents with T1DM incorporate the goals of achieving optimal glycemic control and providing all the essential macro- and micro-nutrients for growth and development. In diabetes camps, a team of registered dieticians specialized in Type 1 Diabetes is responsible for planning and managing meals and snacks [19]. The camp participants are offered three meals and three snacks, two between meals and one before bedtime. Nutritious foods adhering to the Mediterranean diet, with a low glycemic index and high in fiber (fruits, vegetables, whole grains, legumes, nuts, and lean protein from poultry and fish), are preferred [20]. Infringements to planned nutrition can be observed, especially by adolescents, indicating the need for a more flexible and less strict approach, as it may have the opposite results [21].

The portions and type of food are individualized for every child or adolescent according to their gender, height, weight, age, history of hypoglycemic events, and level of physical activity [22]. In case of increased energy demand (e.g., higher levels of physical activity, dehydration, etc.), caloric intake and carbohydrate load are increased accordingly. The medical history and any food allergies are carefully documented upon a participant’s registration to the camp. Every staff member is trained to detect the alarming signs of an allergic or anaphylactic reaction [23].

At diabetes camps, blood glucose (BG) measurements are performed at least four times a day, usually before and after meals, before bedtime, and at midnight. Children and adolescents are encouraged to self-monitor their blood glucose levels, with the camp staff always available to provide support or education when needed [24]. It is common for young children to require assistance in the self-monitoring of blood glucose and performing insulin injections. Either blood glucose meters or continuous glucose monitors (CGMs) can be used [25]. In case of hypoglycemia (BG < 70 mg/dL), a snack containing 15 g of quick-acting carbohydrates is immediately consumed, and glucose levels are re-evaluated in 15 min. Hypoglycemic events are usually more common during the first 48 h of camping, as physical activity increases significantly, and insulin doses have not yet been amended [26].

## 4. Diabetes Camps and Glycemic Control

Glycemic control may be challenging during a diabetes camp attendance, due to remarkable changes in the children’s daily routine compared to home, including nutrition and social and physical activities [27]. Blood glucose levels present fluctuations, while insulin and caloric demand are commonly increased as children and adolescents get involved in multiple activities that affect energy expenditure [28]. Age at T1DM diagnosis definitely affects compliance to insulin therapy, as patients being younger at diagnosis seem to be more mindful during adolescence. Glycemic control is more demanding in adolescents, as adherence to medical instructions is poor [29]. In patients diagnosed during adolescence, it is very difficult to achieve glycemic balance; the diabetes complications of hypoglycemia and DKA are very common in this group [30].

The first study evaluating the metabolic effect of diabetes camps was published in 1984. The study involved 36 children with T1DM participating in a two-week diabetes summer camp. The researchers concluded that glycated serum protein levels were significantly reduced after attendance, with a small decrease in glycated hemoglobin levels, given the short camping duration. Although an individualized meal plan was formed for each participant, the direct effect of nutritional intervention on glycemic control was not analyzed [31].

A Korean study, including 24 children and adolescents, evaluated blood glucose control during a three-day diabetes camp. A healthy diet tailored to the needs of each camping participant was followed. No adverse event of DKA or severe hypoglycemia was observed. Blood glucose levels remained within normal range during the camp period [32]. Another original study compared adolescents with T1DM (*n* = 77) who attended a diabetes camp for 20 days with a control group (*n* = 106). Glycated hemoglobin levels were significantly decreased over time, with the result being preserved at three- and seven-month follow-up. According to parental reporting, adherence to insulin treatment was also increased [33].

A cross-sectional study in a two-day diabetes camp in Indonesia included 28 children and adolescents with T1DM who followed diabetes-specific nutritional planning. The research showed a statistically significant improvement in glycated hemoglobin levels three months after the diabetes camp attendance [34]. Besides, an original study in Cameroon of sub-Saharan Africa, with 32 participants attending a five-day diabetes camp, reported a substantial decrease in glycated hemoglobin levels 12 months post diabetes camp attendance. All participants followed an individualized meal plan [35].

In a five-day diabetes camp with 60 participants with T1DM, medical nutrition therapy was implemented, among other interventions (lectures and games on insulin therapy and dose adjustments, recognition and management of hypo- and hyperglycemia, and management of diabetes complications) [36]. Nutritional knowledge was assessed using a structured questionnaire after the camping days and at the arranged follow-up meetings at three and six months post-attendance. It was shown that nutritional education had a positive impact on glycemic control; however, the result was not preserved at the six-month follow-up. The participants who performed more frequent blood glucose measurements (3–4 times/day) had lower levels of glycated hemoglobin.

In a number of studies, though, the beneficial impact of diabetes camps on glycemic control was not confirmed. A prospective study included 34 participants with T1DM, who received psychoeducative intervention, and 23 controls, who received standard care. The camp lasted eight days, and participants were followed up for 12 months. Despite the implementation of interactive seminars about diet, diabetes-related skills, and educational activities, glycemic control (measured by HbA1c levels) and body mass index did not present a significant change [37]. Additionally, a study with 20 children and adolescents with T1DM, participating in a one-week diabetes camp and receiving an individualized diet plan showed that glycemic control (measured by HbA1c levels) at the three-month follow-up had deteriorated compared to baseline [38]. Another relevant study in 28 adolescents with T1DM attending a 10-day diabetes camp and receiving educational seminars on diabetic nutrition reached the same unfavorable conclusion, having a follow-up at 12 months. Glycemic control was measured using HbA1c levels, too [39]. A possible explanation for this inconsistency in the results between studies involves poor compliance due to economic, social, or cultural problems of participants and is not attributed to the diabetes camp education. Additionally, variability in metabolic status between subjects is a factor inducing heterogeneity to potential comparisons. Poor glycemic control of camp participants before attendance is related to a higher risk for unfavorable results compared to participants with better control, irrespective of the camping interventions, given their short duration.

It is important to note that the majority of children with T1DM are using diabetes technologies, such as insulin pumps (continuous subcutaneous insulin infusion, CSII), CGMs, or hybrid closed-loop systems (artificial pancreas) [40]. According to a previous original study, almost 50% of children attending T1DM diabetes camps are using an insulin pump [41]. Additionally, it has been proved that the use of diabetes technologies significantly improves glucose variability and reduces hypoglycemic events during camp attendance [42]. A study evaluating glycemic control in children and adolescents with T1DM on a hybrid closed-loop system attending a diabetes camp was recently published [43]. As expected, a significant decline in the “Time in Range” was observed during the first 24 h of camping; however, 48 h later, glucose levels returned to the pre-existing normal pattern.

The characteristics of included studies are summarized in Table 1.

The current review presents some strengths and limitations that should be mentioned. The article presents a highly specific aspect of diabetes care. The review’s novelty is enhanced by the fact that, to our knowledge, it is the first literature review evaluating the impact of the nutritional education provided in diabetes camps on the glycemic control of children and adolescents with T1DM. It was shown that the effect of nutritional intervention is generally positive, both at short- and long-term. The basic limitation of the present review, though, is the lack of studies evaluating the direct contribution of nutrition on glycemic control. All the included studies assessed the effect of nutrition among other interventions; thus, the results cannot be attributed exclusively to nutritional education, as multiple factors, acting as confounders, may have significantly impacted the results. Potential confounders include physical activity, close monitoring of glycemic control, medical education lectures, psychological support, and diabetes skills acquired during the camp. Besides, there was heterogeneity in the sample size, the age of the participants, the diabetes camp duration, and the follow-up time points between studies.

## 5. Conclusions

In conclusion, diabetes camps offer significant educational opportunities for children and adolescents with T1DM. However, the deviations from daily routine in the diabetes camp setting may pose challenges to glycemic control and potentially increase the risk of hypoglycemia. These issues are managed effectively due to careful meal planning, close monitoring of glucose levels, and appropriate adjustments to caloric intake or insulin therapy when needed. The majority of the included studies concluded that diabetes camps improve nutritional knowledge providing diabetes-specific nutritional education and supporting healthy eating habits. The favorable effect of diabetes camps on glycemic control appears to be sustained in the short term (<6 months). Thus, continuous nutritional education at regular periods is required to prolong the duration of these beneficial outcomes. Future prospective studies should be conducted to determine the direct impact of nutritional education on the glycemic control of children and adolescents with T1DM attending diabetes camps, taking into account all the possible confounders to reaching safe conclusions. It is suggested that a consensus regarding diabetes camps design (participants, duration, follow-up, etc.) should be adopted to ensure the generalizability of the results.

## Figures and Tables

**Table 1:** Characteristics of included studies.

First Author, Publication Year	Country	Participants	Age (Years)	Diabetes Camp Duration	Follow-Up	Nutritional Intervention	Glycemic Control Measurement	Conclusion
Troncone et al., 2021 [38]	Italy	20	11 ± 0.94	7 days	Baseline & 3 months	Individualized diet plan	HbA1c levels	No beneficial effect on glycemic control
Kang et al., 2017 [32]	Korea	24	13.4 ± 1.7	3 days	None	Healthy diet tailored to the needs of each camping participant	HbA1c levels	Blood glucose levels remained within normal range during the camp period
Dehayem et al., 2016 [35]	Cameroon	32	19 ± 2	5 days	3 & 12 months	Individualized diet plan	HbA1c levels and number of hypoglycemic episodes	Decrease (0.6%) in HbA1c levels at the 12-month follow-up (*p* = 0.029)
Soenggono et al., 2011 [34]	Indonesia	28	7–18	2 days	Baseline & 3 months	Nutrition planning	HbA1c levels	Glycemic control significantly improved (0.51%) at the 3-month follow-up (*p* = 0.004)
Garcia-Perez et al., 2010 [37]	Spain	34	11–18	8 days	12 months	Interactive seminars about diet	HbA1c levels	Glycated hemoglobin and body mass index did not present a significant change
Wang et al., 2008 [33]	USA	77	12–18	20 days	3 months & 7 months	Individualized meal plan	HbA1c levels	HbA1c levels were lower in children who attended the diabetes camp compared to controls at both follow-up time points (*p* = 0.04)
Santiprabhob et al., 2008 [36]	Thailand	60	16 ± 7	5 days	Baseline, 3 months, & 6 months	Lectures on diabetes nutrition and meal planning	HbA1c levels	At the 3-month follow-up there were more patients with HbA1c ≤ 8% compared to baseline; the result was not sustained at the 6-month follow-up
Semiz et al., 2000 [39]	Turkey	28	8–20	10 days	12 months	Education on diabetic nutrition	HbA1c levels	No significant change in glycated hemoglobin levels
Strickland et al., 1984 [31]	USA	36	7–15	14 days	None	Individualized diet plan	Fasting plasma glucose (FPG), HbA1c, and glycosylated serum proteins (GSP) levels	Glycated serum protein levels were significantly reduced (7%, *p* < 0.005)

HbA1c: glycated hemoglobin.
